# Implication of two different regeneration systems in limb regeneration

**DOI:** 10.1002/reg2.16

**Published:** 2014-08-29

**Authors:** Aki Makanae, Kazumasa Mitogawa, Akira Satoh

**Affiliations:** ^1^ Okayama UniversityResearch Core for Interdisciplinary Sciences (RCIS)3‐1‐1 TsushimanakaKitakuOkayama700–8530Japan

**Keywords:** accessory limb model, bone healing, intercalation, limb regeneration, positional values, proximodistal axis

## Abstract

Limb regeneration is a representative phenomenon of organ regeneration in urodele amphibians, such as an axolotl. An amputated limb starts regenerating from a remaining stump (proximal) to lost finger tips (distal). In the present case, proximal−distal (PD) reorganization takes place in a regenerating tissue, called a blastema. It has been a mystery how an induced blastema recognizes its position and restores an exact replica of missing parts. Recently, a new experimental system called the accessory limb model (ALM) has been established. The gained ALM phenotypes are demanding to reconsider the reorganization PD positional values. Based on the ALM phenotype, it is reasonable to hypothesize that reorganization of positional values has a certain discontinuity and that two different regeneration systems cooperatively reorganize the PD axis to restore an original structure. In this review, PD axis reestablishments are focused on limb regeneration. Knowledge from ALM studies in axolotls and *Xenopus* is providing a novel concept of PD axis reorganization in limb regeneration.

Urodele amphibians, such as axolotls and newts, can regenerate their limbs after amputation. Cells appear from the amputation surface and form a blastema. A regeneration blastema consists of undifferentiated cells called blastema cells. The blastema cells are a heterogeneous population. Some are lineage‐committed and some are multipotent or at least bipotent (Kragl et al. 2009; Hirata et al. 2010). The induction process in such blastema cells is interesting and worthy of study. However, the present review focuses on mechanisms by which blastema cells recognize their location and reestablish their positional values to regenerate missing parts.

## The accessory limb model as a unique study system for reestablishing positional values in a blastema

**Figure 1:**
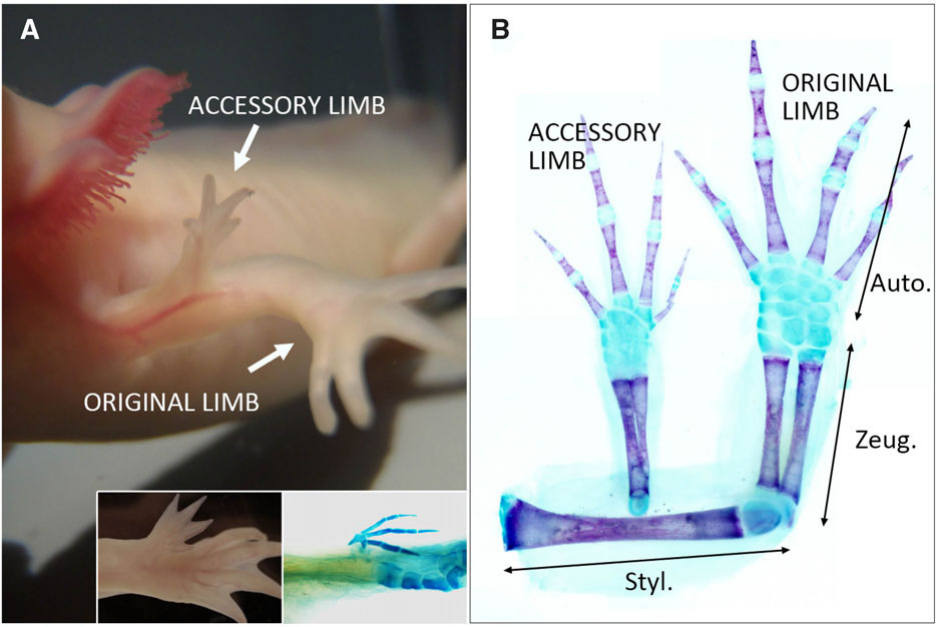
Accessory limb formation in axolotls. (A) A typical phenotype of accessory limb induction in the stylopod. (A, insets) Accessory limb induction in the zeugopod region. Autopod part was induced. Right is the Alcian Blue staining to visualize the skeletal pattern. (B) Cartilage was visualized by Alcian Blue and calcified bone was visualized by Alizarin Red. Auto., autopod; Zeug., zeugopod; Styl., stylopod.

The accessory limb model (ALM) was established in axolotls as an alternative experimental system for limb regeneration (Maden & Holder, 1984; Endo et al. 2004). ALM permits the study of limb regeneration without amputation (Makanae & Satoh 2012). A representative phenotype of the ALM is shown in Figure 1. To induce an accessory limb, skin wounding, skin grafting from the contralateral side and nerve deviation are necessary. In this process, basically no damage is done to deeper tissues such as muscles. Such surgical procedures induce the development of an additional limb from the original limb (Fig. 1). ALM is a powerful experimental system for studying blastema induction mechanisms (Makanae & Satoh 2012) and proximodistal (PD) axis reorganization.

**Figure 2:**
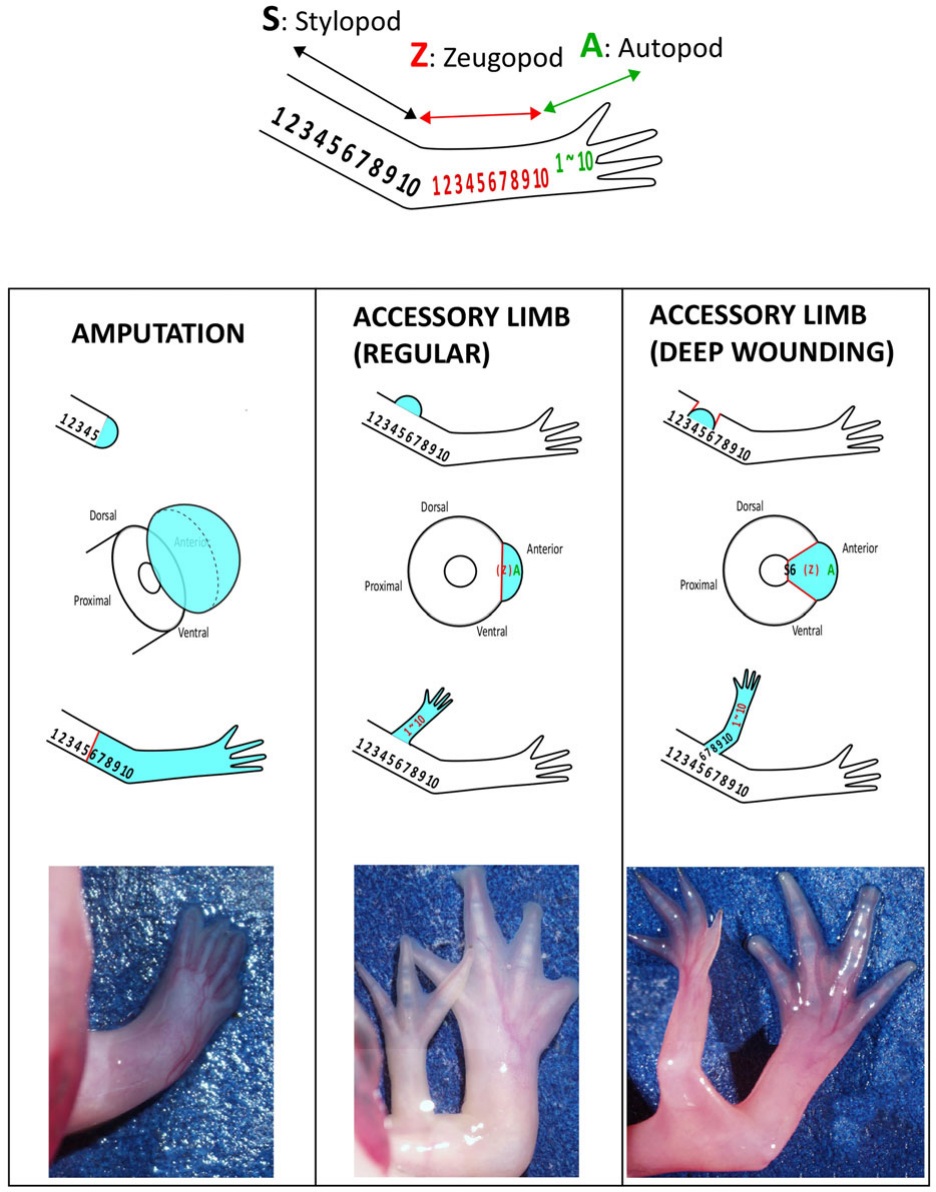
Proximal−distal reorganization in the ALM. Top: Conceptual positional values. S, stylopod; Z, zeugopod; A, autopod. Numbers 1−10 are assigned from proximal to distal. Left column: Regeneration in an amputated limb. Stylopod is amputated at the S5 position and S6−10 are restored by a regular limb regeneration process. Middle column: Accessory limb induction in a stylopod region. Basically the limb surface (skin) is damaged and a blastema is induced on the surface of the limb. A blastema acquires A+Z values, resulting in lower limb formation. Right column: An accessory limb with half stylopod. Wounding reaches a stylopod bone and a blastema is induced on the damaged bone. The induced blastema contains S6−10 values and regenerates an accessory limb with half stylopod.

In regular ALM surgery, an accessory limb is induced within a stylopod area (Fig. 1A, B). Within this area, an induced limb possesses only autopod and zeugopod parts and no stylopod part (Satoh et al. 2010b). Even though an accessory limb is induced in a relatively proximal region of a stylopod, the induced limb has only autopod plus zeugopod (Fig. 2, middle column). This finding suggests that a blastema that is induced within the stylopod segment acquires only “zeugopod + autopod.” Indeed, a blastema appears to obtain distal information from stump tissues. When an accessory limb is induced within a zeugopod area, only a distal structure, in the form of autopod parts, is reconstituted (Fig. 1, insets) (Satoh et al. 2010b). These results suggest that the PD positional values received by an induced blastema are only distal values, excluding any values within the segment where an accessory limb is induced. Thus, the phenotypes of the ALM are useful for investigating the reestablishment of positional values.

## Reorganization of PD positional values in limb regeneration

Intercalation is the primary principle of organ regeneration (French et al. 1976; Agata et al. 2007). On this principle, distal information is established first, and then the established distal part(s) interact with the remaining proximal part(s) to create an “intermediate” part. Such intercalary positional establishment appears to be not only unique to limb regeneration but also a general way of organizing limb formation (Shimizu‐Nishikawa et al. 2003; Nakamura et al. 2007; Mariani et al. 2008). Recently, PD axis reconstitution in limb regeneration has started being studied at the molecular level (Mercader et al. 2005; Roensch et al. 2013). Such molecular data may demand a reconsideration of intercalary limb regeneration. However, it is necessary to accumulate more molecular evidence in order to find consistency with the current intercalary theory. In this review, skeletal phenotypes of ALM are focused upon since the phenotypes of ALM appear to be providing important insights into intercalary regeneration.

ALM phenotypes indicate that a blastema can be obtained from “distal” information without “proximal” information. For explanation, numbers and letters (S1−10, Z1−10, and A1−10), which have historically been used to describe limb regeneration, are represented in Figure 2. Also detailed information and skeletal patterns of ALM are described in our previous paper (Satoh et al. 2010b). In accordance with the principle of intercalary limb regeneration, blastema cells acquire distal value(s) and begin to interact with stump tissues to create intermediate structures. An exact replica of the original structures is then regenerated. If a stylopod is amputated at a middle position, half of a distal stylopod is invariably regenerated (Fig. 2, left column). In this case, a blastema is formed and reconstitutes S6−10, Z1−10, and A1−10. For this reason, it is believed that a blastema reestablishes positional values from the exact position of an amputation. However, ALM phenotypes give us a chance to reconsider this notion. As mentioned above, an accessory limb in a stylopod region contains no stylopod part(s) even when an accessory limb is induced in the middle region of a stylopod (Fig. 1A, B, Fig. 2, middle column) (Endo et al. 2004; Satoh et al. 2010b; Makanae & Satoh 2012). This finding implies that a blastema acquires A and Z values and that the stump does not transmit the information of S6−10 to the induced blastema (Fig. 2, middle column). Similarly, when an accessory limb is induced in a zeugopod, the only values established in a blastema are A1−10, resulting in regeneration of only the autopod (Fig. 1A, insets). In theory, the ALM blastema should receive half of the S or Z values, given that ALM blastemas are induced in the middle of each segment (S5 or Z5 position). It is thus somewhat surprising that a blastema reestablishes only distal positional values. We hypothesize that information from stump tissues is to establish numbers from the next segments, not including any information to establish numbers within the segment where limb amputation is performed.

What happens to half of the proximal values when a blastema is induced in the middle of a stylopod? These missing S6−10 values can be generated using the ALM procedure with deep wounding, resulting in an accessory limb with a half stylopod (Fig. 2, right column). In regular ALM surgery, only skin and not deeper tissues are damaged (Fig. 2, middle column). To induce S6−10, a wound sufficiently deep to damage a stylopod bone is created by the ALM surgery (Fig. 2, right column). In this case, the regenerated half stylopod bone (S6−10) is directly extended from the stump stylopod bone (Fig. 2, right column). Indeed, proximal regeneration depends upon damaging the stump bone.

**Figure 3:**
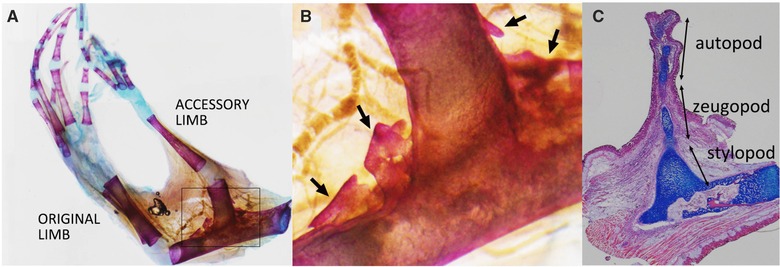
Histological observation. (A), (B) Skeletal pattern was visualized by Alizarin Red and Alcian Blue. (B) Higher magnification of (A). Some ectopic bone formations can be seen in the proximal region. Such ectopic bone particles cannot be seen in the distal region. (C) Hematoxylin, eosin, and Alcian Blue staining. The regenerating half stylopod consisted of relatively fat cartilage compared with cartilage in the distal region.

Proximal regeneration in the ALM shows characteristic features. Ectopic bone particle(s) are often observable around the regenerated stylopod (Fig. 3A, B). Limb regeneration, particularly in pattern formation processes, has been proposed to mimic limb developmental processes (Muneoka & Bryant 1982; Bryant et al. 2002). Such ectopic bone particles are thus characteristic, given that the developmental (genetic) program does not usually allow the production of such ectopic structures. Moreover, the process of proximal regeneration also implies distinct regeneration from the distal region (Fig. 3C). Histological observation of the regenerating accessory limb with regeneration of the proximal structure is shown in Figure 3C. Distal structures are regenerating and some skeletal elements can be observed (Fig. 3C). In the proximal region relatively fat cartilage extension can be observed compared with the distal ones (Fig. 3C). Later, the wide and extending cartilage is reshaped into the proper size of the stylopod and cartilage elements are replaced by calcified bone. Some small bone particles can be observed around the proximal region of the induced stylopod (Fig. 3B). Such reshaping and ectopic bone particles are not observed in normal limb development/regeneration, suggesting that proximal regeneration is independent of a blastema that drives developmental programs and is dependent on another regeneration system.

This hypothesis of blastema‐independent regeneration in a proximal region is supported by another experimental result. A hand part was dissected and grafted onto a limb amputated at the stylopod level. Blastema formation was inhibited because the amputated surface was occupied by the hand graft, indicating that there was no room for blastema formation. Although no apparent blastema is formed in this situation, the proximal part (stylopod) is restored (Bryant & Iten 1977; Satoh et al. 2010b). The amputated stylopod was extended distally and segmentation occurred between the hand graft and the extended stylopod. This result supports the hypothesis that a distinct regeneration system from blastema formation plays a role in proximal regeneration.

**Figure 4:**
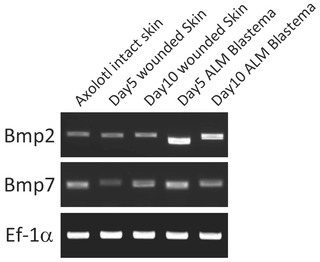
Blastema expresses Bmp2 and Bmp7. Bmp2 and Bmp7 expression was investigated by reverse transcription polymerase chain reaction (PCR). Ef‐1a is an internal control. Samples were prepared as indicated. PCR was performed by Takara ExTaq. The number of PCR cycles is 30.

Stylopod damage should lead to bone healing responses. In the ALM, bone healing responses may explain stylopod regeneration. As the stylopod bone is damaged, the bone healing process is activated. In the bone healing process, chondrocytes and perhaps osteoblasts proliferate in the damaged region. The ALM blastema is then formed on top of the bone healing region and establishes “distal” A and Z positional values (Fig. 2, right column). Bone healing chondrocytes and some associated cells recognize the ALM blastema as distal and bone healing progresses toward the newly established distal parts. It is possible that a newly formed blastema can be a source of bone morphogenic proteins (BMPs) since at least Bmp2 and Bmp7 are expressed in the blastema in ALM (Fig. 4). Such BMPs from the blastema probably direct bone healing. Such directed bone healing is expected to be one of the forces of proximal regeneration.

## Consistency with previous results

**Figure 5:**
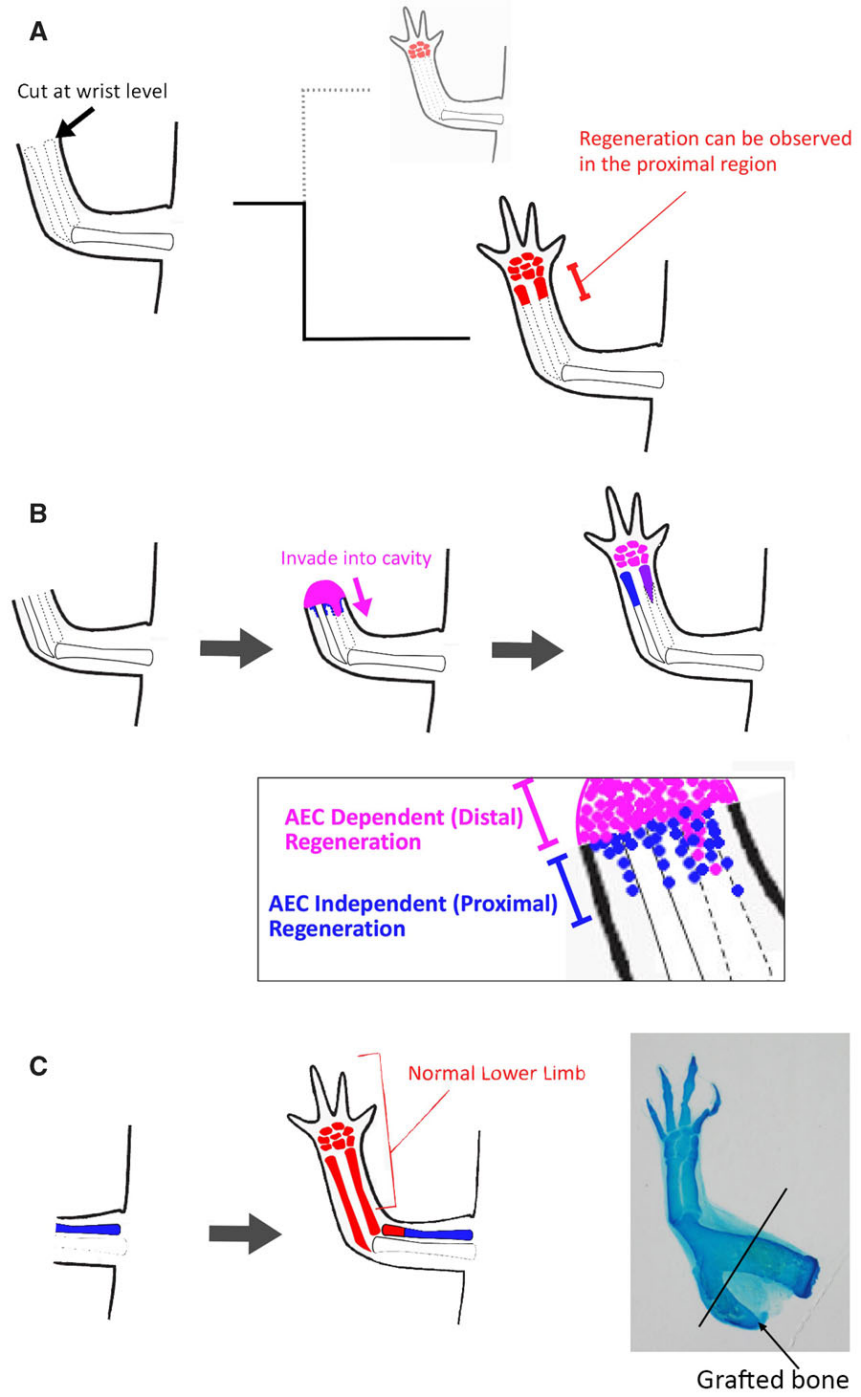
An interpretation of previous experimental results. The illustration is drawn on the basis of the results reported by Goss (1956). (A) Both ulna and radius were removed and the limb was amputated at the wrist level. Because amputation was achieved at the wrist level, only autopodial elements should have been regenerated in theory. However, partial zeugopodial regeneration took place. (B) An interpretation of Goss's experiment. The ulna was extirpated and the limb was amputated. A partial ulna was regenerated. The experiment in (A) suggests that blastema cells can invade into a cavity and participate in proximal regeneration. Therefore, it is likely that blastema cells invade into a cavity and regenerate partial ulna. (C) An ulna was added to the stylopod and the limb was amputated. The added ulna was regenerated but distal parts showed a normal skeletal pattern without any duplicated skeletal elements. When a humerus was engrafted, the same phenotype was obtained (inset). The humerus was grafted as indicated in Goss (1956). The regenerated humerus was fused to the other. This fusion was also reported by Goss (1956). The regenerated distal parts were normal (*n* = 4/4).

Goss reported many insightful experimental results (Goss 1956). One of his famous experiments involved extirpating the ulna and amputating the ulnaless limb in the middle region of the lower arm (Fig. 5B). He found that the amputated limb regenerated the lower arm, including the half ulna (Fig. 5B). This finding appears inconsistent with the above statement that a blastema reconstitutes only the distal part(s). However, Goss also proposed the possibility of invasion of blastema cells into spaces previously occupied by bone. His results consistently support the idea of invasion of blastema cells. For example, the ulna and radius were completely extirpated and the limb was amputated at the wrist level (Fig. 5A). Theoretically, a blastema would regenerate the distal parts from the wrist. However, the blastema regenerated the distal parts and also the small ulna and radius, proximal structure to the amputation site (Fig. 5A). This result suggests that blastema cells can invade into proximal spaces, consistent with the observation that blastema cells can participate in bone healing when they are placed in the bone healing region (Satoh et al. 2010a). Given that blastema cells can adapt to their surrounding environment, it is very likely that invading blastema cells recognize the surrounding environment and form zeugopodial elements via proximal regeneration.

Another experiment by Goss helps in understanding PD axis reconstitution. An ulna was additionally grafted into the stylopod region near the humerus (Fig. 5C) and the limb was amputated through the graft bone and the humerus. Distal structures regenerated normally (Fig. 5C). When the humerus instead of the ulna was used for grafting, the same result was confirmed (Fig. 5C). These results support the idea that distal and proximal regeneration are controlled differently. However, without molecular evidence, other interpretations are still possible. Molecular analysis should be performed to bring ALM knowledge into accord with the former results.

## PD axis reorganization in the ALM in *Xenopus laevis*

**Figure 6:**
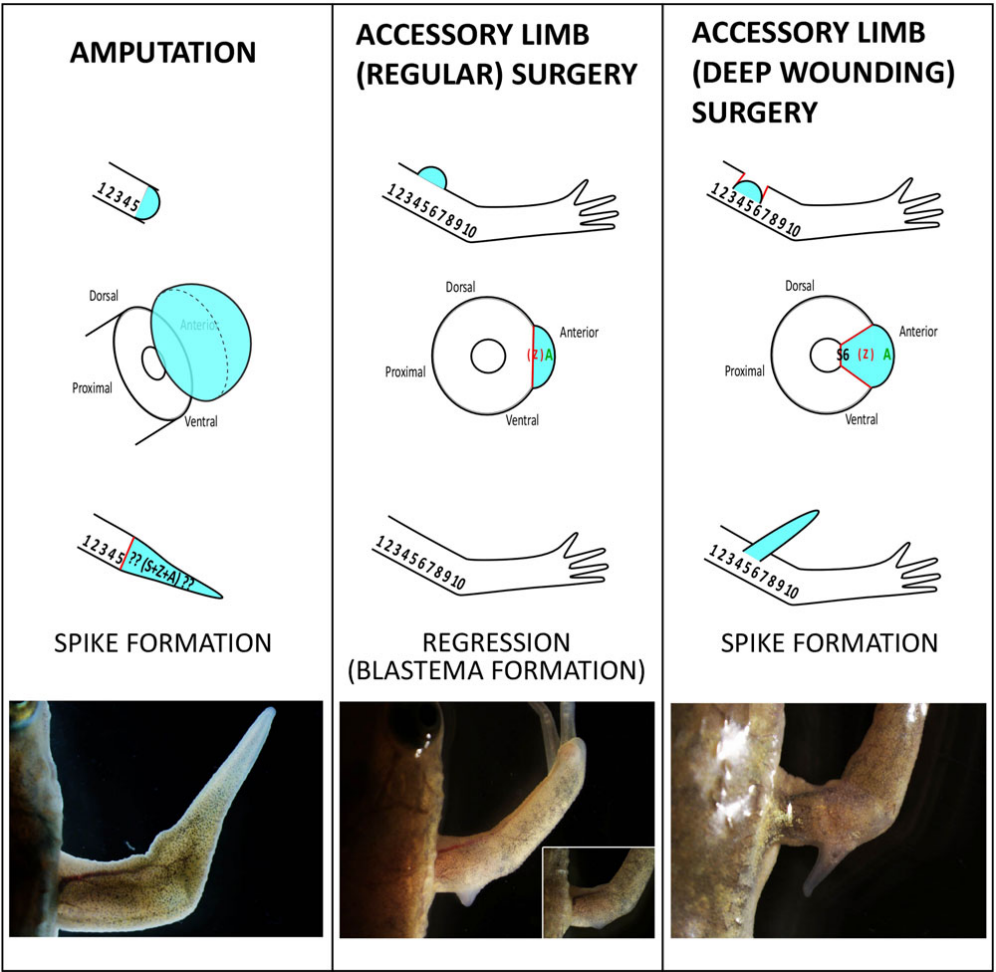
Xenopus limb regeneration and ectopic blastema formation. Left column: Regeneration in an amputated limb. Stylopod is amputated at the S5 position. Hypomorphic structure, called a spike, is induced. Middle column: Accessory blastema induction in a stylopod region. All nerve bundles are rerouted to the skin wound, leading to an ectopic blastema formation. However, the induced blastema cannot keep growing and shrinks at last (inset). Right column: An accessory spike formation. Wounding reaches a stylopod bone and a blastema is induced on the damaged bone. The induced blastema forms a cartilaginous spike. In the picture spike and blastema are induced in the posterior region, not the anterior region, for experimental reasons.

*Xenopus* frogs cannot regenerate limbs but can grow a hypomorphic cartilaginous structure called a spike (Fig. 6, left column) (Dent 1962; Endo et al. 2000). Because of its ability to grow a spike distally, *Xenopus* frog limb regeneration can be considered as an intermediate regeneration phenomenon between non‐regenerative and regenerative animals. The blastema formation is dependent on nerve presence (Endo et al. 2000; Yokoyama et al. 2011) because denervation of a frog limb results in a failure of blastema induction. Such nerve dependence is similar to that in axolotl/newt limb regeneration. Even though a frog blastema has nerve dependence as in urodele amphibians, whether a frog blastema is a blastema is disputed, given that a frog blastema cannot form a patterned limb. However, a frog blastema shows reactivation of some developmental genes, including genes related to limb PD axis establishment. For instance, HoxA11 and HoxA13 were reported in a frog blastema (Ohgo et al. 2010) and Fgf8, which is an apical ectodermal ridge (AER)/apical epithelial cap (AEC) marker gene, is expressed in a distal blastema epithelium. Given the reactivation of some developmental genes along the PD axis, a frog blastema attempts to rebuild the PD axis, though incompletely. Thus, a cartilaginous spike may be considered as a hypomorphic structure with a certain level of reestablished positional values, whether these values are complete or incomplete.

ALM surgery is possible, and accessory structures are inducible in *Xenopus laevis* (Mitogawa et al. 2014). An accessory blastema can be induced by skin wounding plus nerve deviation as in axolotl ALM surgery (Fig. 6, middle column). The induced blastema expresses some blastemal genes but cannot continue growing. The induced accessory blastema cannot maintain its growth and finally begins to regress (Mitogawa et al. 2014). With deep wounding plus ALM surgery, which causes proximal regeneration in the axolotl ALM as described above, an accessory blastema is inducible and a cartilaginous spike is formed (Fig. 6, right column). Therefore, spike appearance is associated with deep wounding. In the axolotl proximal regeneration in ALM, directional bone healing appears to play an important role, suggesting that the growing cartilaginous spike in *Xenopus* is associated with bone healing, that is, similar to proximal regeneration in the axolotl ALM. Nerve interaction with overlying epithelium gives rise to a blastema having distal information, and this blastema may lead the bone healing cells in the newly induced distal direction. Otherwise, an induced blastema works as a BMP source, which has mitogenic ability for cartilage cells and persists at the top of the bone healing region. This continuous and one‐way input may result in directional cartilage extension, an effect that remains to be clarified. Thus, the comparison of ALM phenotypes in *Xenopus* and axolotl provides interesting insights into limb regeneration.

Successful blastema induction in the *Xenopus* ALM strongly suggests that nerve functions and fibroblasts growth factor (FGF) and BMP signaling in blastema induction are similar or identical to those in urodele amphibians. Both signaling activations are expected since Fgf8 is expressed in *Xenopus* frog blastema and Bmp genes have also been detected (Endo et al. 2000; Mitogawa et al. 2014). Subsequent events must be responsible for the differences in regeneration ability between urodele and anuran amphibians.

## Digit/limb reorganization in mice

Mice can regenerate their digit tips without special medical treatment (Borgens 1982; Han et al. 2003). When the very tip of a terminal phalange is amputated, a blastema is formed and regenerated. However, a more proximal amputation, even in the terminal phalange, does not result in successful regeneration. A blastema induced on the amputated digit tip shows somewhat different features from those in amphibians (Muneoka et al. 2008). For example, there is no report of AER/AEC establishment in the blastema or of nerve dependence. Thus, it may be difficult to compare a blastema in urodele amphibians with a mouse digit blastema side by side. However, it is also true that expression of a few common genes is detectable in both cases. For example, Msx1 and Msx2 are transcriptional repressors expressed in mouse digit tip regeneration (Han et al. 2003; Muneoka et al. 2008). Mouse digit tip regeneration studies are in their early stages compared with limb regeneration studies in amphibians. Further investigation should reveal analogies between limb regeneration in mice and amphibians.

Although very limited information regarding mouse limb regeneration is available, a strong regenerative inducer has been found. Digit regeneration does not occur after amputation proximal to the distal regions of the terminal phalanx. BMP2 application can promote digit regeneration responses after amputation at the non‐regenerative level. However, such promotion of regeneration ability by BMP application can be observed within a terminal phalanx (Yu et al. 2010). BMP2 application can promote bone extension when a digit is amputated in the second phalanx, but the induced regenerate does not create a joint (Yu et al. 2012). Such segment‐specific skeletal regeneration by BMP application can be seen in actual limb amputation (Ide 2012; Yu et al. 2012). Therefore, regenerative responses induced by BMP application cannot include the regeneration of a distal structure.

## Intra‐ and inter‐segmental regeneration

Regeneration in axolotl and *Xenopus* ALMs and mouse digit regeneration imply the existence of two distinct regeneration systems: (1) regeneration that creates a distal structure over a damaged segment (inter‐segmental regeneration); and (2) regeneration that occurs within a damaged segment (intra‐segmental regeneration). This concept was originally proposed by K. Muneoka (Tulane University, New Orleans, LA). Inter‐segmental regeneration is likely to be related to AEC formation (blastema independent mechanism) and intra‐segmental regeneration to be AEC‐independent (blastema independent mechanism). AEC is induced by regular ALM surgery and such AEC induction in the ALM results in regenerating distal structures. In other words, inter‐segmental regeneration is induced by AEC formation, and proximal regeneration can be observed without AEC formation. Grafting a hand onto an amputated stylopod results in stylopod regeneration without regeneration of zeugopodial structures (Bryant & Iten 1977; Satoh et al. 2010b), indicating that intra‐segmental regeneration is blastema independent. Furthermore, intra‐segmental regeneration is associated with bone healing response. Proximal regeneration can be observed in the ALM with deep wounding as mentioned above. This interpretation is possible even in the *Xenopus* ALM. The *Xenopus* ALM without deep wounding may induce inter‐segmental regeneration, but the induced blastema cells may not be sufficiently dedifferentiated or maintained, leading eventually to the disappearance of the induced structure. *Xenopus* ALM surgery with deep wounding induces bone healing responses since the stylopod bone has been damaged. Nerve−wound epithelium (WE) interaction gives rise to AEC formation leading to blastema formation. The induced ALM blastema expresses BMPs (Mitogawa et al. 2014), suggesting that continuous BMP sources can be expected in the newly established blastema having “distal” values. Thus, intra‐segmental regeneration may be directed by a continuous BMP source(s). In mouse digit regeneration, AEC formation has not been reported and is very unlikely to be induced. Amputation at the regenerative level leads to BMP reactivation and amputation at the non‐regenerative level does not (Han et al. 2003). Furthermore, BMP supplementation of non‐regenerative amputation gives rise to regeneration within a segment (intra‐segmental regeneration). Based upon these observations, we hypothesize that there are two distinct regeneration systems. ALM experiments and mouse digit regeneration studies can be linked by the concept that inter‐segmental and intra‐segmental regeneration work cooperatively to restore the original structures.

Inter‐ and intra‐segmental regeneration are probably coordinated mutually because a regenerated limb shows no obvious boundary between the two modes of regeneration. Thus, each regeneration system should recognize the other, and the two should coordinately regenerate the original structure. FGF signaling may play a role in coordination between the two regeneration systems, but a definitive explanation for how the two systems are coordinated is lacking. FGF signaling is a mediator of intercalary responses in mouse and *Xenopus* (Shimizu‐Nishikawa et al. 2003; Mariani et al. 2008). As mentioned above, hand grafting onto an amputated stylopod results in stylopod regeneration but no intermediate structure (zeugopod). FGF2 application between the hand graft and stump tissues induced intermediate structures (zeugopod) (Satoh et al. 2010b). These findings suggest that FGF signaling coordinates PD organization by mediating intercalary responses. However, elucidation of the coordination mechanisms between inter‐ and intra‐segmental regeneration awaits further study.

## Conclusion

Although limb regeneration has been believed to be one system, ALM studies suggest that there are at least two distinct systems, which should be considered when interpreting results obtained from limb amputation experiments.
